# Optimization of Mechanical and Thermal Properties of iPP and LMPP Blend Fibres by Surface Response Methodology

**DOI:** 10.3390/polym10101135

**Published:** 2018-10-12

**Authors:** Sohail Yasin, Danmei Sun, Hafeezullah Memon, Feichao Zhu, Han Jian, Yu Bin, Ma Mingbo, Munir Hussain

**Affiliations:** 1 School of Textiles and Design, Heriot-Watt University, Galashiels TD1 3HF, UK; d.sun@hw.ac.uk; 2 Key Laboratory of Textile Science and Technology, Donghua University, Shanghai 201620, China; hafeezullah_m@yahoo.com; 3 School of Materials and Textiles, Zhejiang Sci-Tech University, Hangzhou 310018, China; zhufeichao1988@126.com (F.Z.); hanjian8@zstu.edu.cn (H.J.); yubin7712@163.com (Y.B.); mamingbo@zstu.edu.cn (M.M.); munir88@zju.edu.cn (M.H.); 4 Department of Polymer Science and Engineering, Zhejiang University, Hangzhou 310027, China

**Keywords:** polypropylene, low molecular low modulus polypropylene, RSM, optimization

## Abstract

Optimization of the mechanical and thermal properties of isotactic polypropylene (iPP) homopolymer blended with relatively new low molecular low modulus polypropylene (LMPP) at different blend ratios was carried out via surface response methodology (RSM). Regression equations for the prediction of optimal conditions were achieved considering eight individual parameters: naming, elongation at break, tensile strength and elastic modulus, crystallization temperature (*T*_C_), first melting temperatures (*T*_M1_), heat fusion (*Hf*), crystallinity, and melt flow rate (MFR), which were measured as responses for the design of experiment (DOE). The adjusted and predicted correlation coefficient (R^2^) shows good agreement between the actual and the predicted values. To confirm the optimal values from the response model, supplementary experiments as a performance evaluation were conducted, posing better operational conditions. It has been confirmed that the RSM model was adequate to reflect the predicted optimization. The results suggest that the addition of LMPP into iPP could effectively enhance the functionality and processability of blend fibres if correctly proportioned.

## 1. Introduction

Isotactic polypropylene (iPP) polymer is a type of broadly utilised polymer that is used for its low cost and appealing mechanical properties, but only when blended with other polymeric materials, as iPP alone has some drawbacks. For example, brittleness at low temperatures [1], low impact toughness, development of static electricity and poor bonding of hydrophilic reactive groups in iPP chains [2]. Mixing iPP with other elastomeric polymer materials evades these drawbacks, enhances the effective protection and broadens its applications. Moreover, due to the higher strength and modulus of crystalline materials, addition of polypropylene (PP) to the elastomeric matrix materials are expected to improve the processability, higher modulus and enhance the chemical resistance, while keeping high abrasion resistance, tear strength and flexibility, and shock-absorbing properties [3]. In literature, papers can be found on PP blends to control the rheology, crystallinity, spherulite structure and so on [4]. However, the main focus of the research can be found on chemical modification of iPP in order to expand the applications by generating value-added materials with improved mechanical and thermal properties. It is rare to find theoretical and experimental investigations on crystallization control of iPP, which is very important for stretchability. Kanai et al. controlled the crystallization speed of iPP with low molecular low modulus polypropylene (LMPP), which has low crystallinity and low melt temperature [4]. Moreover, such PPs with high molecular weight and low isotacticity are synthesised with certain doubly bridged metallocene complexes [5]. Due to its unique elastomeric properties and compatibility with iPP, LMPP can be applicable to hot melt adhesives, elastic fibres, nonwoven fabrics and so on [5].

Polymers are mainly blended with other polymeric materials to combine existing polymers into new compositions obtaining specific properties, which also allows the faster development of new materials. In polymer blending, miscibility is the most important property, which also refers to the solubility when two polymers dissolve in each other at the right proportion [6]. Sometimes polymer blends with one component in suspension as dispersed droplets display ‘fractionated crystallization’ as a result of crystallization [7]. Whereas, the immiscibility is a restraining factor for the production of various blend polymers. Consequently, compatibilization agents are essential for their production. Melt flow rate (MFR), which is the processability of a polymer, is considered important in PP manufacturing; whereas, PP products with high MFR are easy to process and also boost production speed [8]. Li et al. also reported that highly desirable iPP with high MFR is schematised for production by 2025 in China. The current state of the art methods of producing PP products with good flowability is direct polymerization and degradation by peroxide. However, these two methods are hurdled by limitations, for instance, chemical agents used in the degradation process affects the quality of the final PP products and also peroxide compounds have an unbearable smell [8]. To cope with such issues, it is important to apply modern technologies and find alternate procedures for desired polymers with higher MFRs.

The attributes of blend iPP, such as morphology, crystallinity and crystallization control are dependent mainly on the blended components. On one hand, with extensive theoretical and experimental investigation on the miscibility of polymer blends, the nature and characteristics of the amorphous/amorphous or amorphous/crystalline polymer blends are well accepted [9,10]. However, it is difficult to predict optimised iPP blending, as many morphological complications are reported when blended with different polymers with crystal–crystal interactions and amorphous–amorphous interactions, or with an unclear understanding of their miscibility [10]. Therefore, when blending, it is important to distinguish the correlation amongst individual microstructures, material processing and optimization properties that enable the materials to achieve the desired properties [11]. Moreover, due to several factors, the scale-up viability of relatively new polymers has started to become difficult, and the optimal operating condition is one of them. Consequently, computational modeling to assist process optimization has become increasingly popular, which not only minimises the time investment but also resources for the experimental work [6,11].

To achieve high performance and productive polymeric fibre, the optimization of the fabricating process is a key factor to be considered. Therefore, the aim of this research is to employ a computational and statistical tool, a response surface method (RSM) as a design of experiment (DOE), and to optimize the processability of fibres by predicting blend ratios of iPP and LMPP (a relatively new polymer). The considered variables are thermal and tensile properties, in order to have a better understanding of LMPPs effects on iPP blend fibres.

### Response Surface Methodology

In recent years, RSM has been widely used for establishing the optimum input parameters in different manufacturing applications, especially in the domain of textiles and polymeric materials. For instance, it has been used in maximizing processing parameters of chemical finishing of flame retardant textiles [12], eco-friendly textile finish [13], antibacterial silk fabric [14], biodegradable plastic pellets by melt extrusion [15], enhance propylene polymerization [16], mechanical finishing to improve properties of PP composites [17], adhesion of PP based extrusions [18], and so on. Compared to other statistical experimental design methods, RSM reduces the number of experiment trials, which are needed to assess various parameters and their interactions while building models [19].

The conditions for the optimal DOEs are mostly related to the mathematical modelling of a process and, usually, these mathematical models are polynomials having unknown structures, and consequently the corresponding experiments are designed for particular problems only. Moreover, choice of DOEs can largely influence the building of a response surface and accuracy of the approximations. At first, RSM was developed to model experimental responses [20,21], later migrated into the modelling of numerical experiments. In physical experiments, imprecisions are due, for instance, to errors in measurement or numerical noise in results, while RSM applied to the design for optimization reduces expensive analysis methods and their associated numerical noise [22]. Theoretically, RSM is carried out in three phases [23,24]. (a) Experimental design: Every single parameter is kept constant, while exploring the impacts of independent variables on the design framework. Sometimes, it is not possible to consider all the independent experimental parameters. Here, for instance, different polymer melting temperatures (first and second melting points), spinning conditions, etc. can complicate and make it difficult to replicate the design if performed with different experimental conditions. (b) Mathematical modeling: It is important to build a mathematical model which correlates with the experimental results. RSM models a relationship between input parameters and their response variables. (c) Model confirmation: It is important to investigate model adequacy check in order to explain the relationships between input-response variables. Also, a model should be validated and adequate enough to make further predictions and estimations. Bezerra et al. have suggested following stages for the application of RSM as an optimization technique, which are: (1) screening of variables; (2) choice of the experimental design; (3) mathematical–statistical treatment of the data; (4) evaluation of the model’s fitness; (5) determination of the optimal conditions; and (6) obtaining the optimum values [25]. Although, in a similar context, to explore the optimization of experimental conditions, RSM is found to be exhaustive but not limited to the phases or stages mentioned.

## 2. Materials and Methods

### 2.1. Materials and Sample Preparations

For the blending, iPP, S2040 (Ziegler–Natta) was provided by SECCO Petrochemical Limited, Shanghai, China, with an average molecular weight of 2.0 × 10^5^ g/mol. LMPP (LMPP S901) were provided by Idemitsu Kosan Co., Ltd., Tokyo, Japan. The blend mixing ratios of iPP/LMPP (*wt*/*wt*) were used according to the lower and upper limit of DOE to get RSM responses. The basic molecular characteristics of the individual iPP and LMPP are presented in Table 1.

The iPP/LMPP blend fibres at different compositions were prepared after blending the two polymers for 2 min at room temperature with the help of a mechanical blender, made by Giant Co., Ltd., Kunshan, China. Homopolymers were blended with a twin-screw extruder (TSE-30A Ruiya extrusion system Co., Ltd., Nanjing, China) for melt blended samples with an L/D value of 40, where the processing speed and temperature were 60 rpm and 210 °C respectively. The samples were then cut into pellet form after drawing.

The iPP/LMPP melt-spun fibres were prepared with a rheometer accessory (Haul-off drafting device) (Rosand RH7, Malvern, Worcestershire, UK) at room temperature 25 °C. The spinning temperature was set at 190 °C, and the drawing and collecting roller speed was 30 m/min. The iPP/LMPP monofilaments were prepared with a linear density of about 0.7 dtex. Other preoperational and experimental details can be found in previous studies [1].

### 2.2. Characterization of Mechanical and Thermal Properties

The mechanical attributes of the melt-spun fibres produced were characterised using an Instron 2365 universal testing machine. The testing conditions of iPP/LMPP filament were carried in a standard conditioning lab at a temperature of 25 °C ± 2 °C and relative humidity 62–68%. For the filament mechanical test, the gap between two jaws was set at 10 cm and filaments were extended following the movement of the upper jaw set at a speed of 100 mm/min.

The melting behavior and crystallization of iPP/LMPP were measured with a Perkin Elmer DSC 8000 differential scanning calorimeter (Perkin Elmer, Waltham, MA, USA), with an intercooler lowermost temperature of −90 °C. The temperature of iPP/LMPP samples was calibrated with pure Indium (*T*_M_ = 156.6 °C). Samples of 5 mg were loaded onto the sample pan, from room temperature to 200 °C under a nitrogen atmosphere. The first heating rate 10 °C/min was used, whereas isothermal holding was set at 200 °C for 3 min, and then the samples were heated again to 200 °C with heating rate of 10 °C/min.

iPP and LMPP blend fibre samples were measured for their thermal properties, namely crystallization and melting behaviour, with a Perkin Elmer DSC 8000 differential scanning calorimeter (Perkin Elmer, Waltham, MA, USA), which has an intercooler lowermost temperature of about −90 °C. Temperature of the fibre samples was calibrated by using pure Indium (*T*_M_ = 156.6 °C). Five milligrams of the sample was cut from the blended specimen and loaded into the sample pan, from room temperature to 200 °C at the heating rate of 100 °C/min in nitrogen atmosphere. The isothermal holding was at 200 °C for 3 min in order to erase the thermal history, followed by cooling at a rate of 50 °C/min to −30 °C to keep the amorphous part, in order to make the glass transition temperature (*T*g) more obvious. The area of the exothermic curve and temperature of the peak were taken as the crystallization temperature (*T*_C_). As the temperature reached −40 °C, it was reheated again at a rate of 10 °C/min and the melting thermogram was measured. *T*g and temperature of the peak and the area of the endothermic curve were taken as the melting temperature (*T*_M_), and the heat of fusion, (△*Hf*), respectively.

MFR was calculated using RL-Z1B1, provided by Hass Jorda Scientific Instrument Co., Ltd., Shanghai, China. The test was repeated five times at a temperature of 230 °C, load 2.16 Kg and warm-up time 2 min, and the average result was used.

Morphology of the pure iPP and iPP/LMPP blend fibres was carried out with a JSM-5610LV scanning electron microscope (JEOL Company, Akishima, Tokyo, Japan). Since pure LMPP has a low melting point and low melting strength, it could not be drawn into fibres, therefore its fibre SEM was not feasible. However, morphological studies of pure LMPP in molten and film form can be found elsewhere [1]. All the sample fibres were coated with gold prior to scanning. The fibres’ surface was investigated with secondary electrons of 5 kv to get SEM images.

### 2.3. Methods

Thermal and tensile properties with eight individual parameters, namely elongation at break, tensile strength and elastic modulus, crystallization temperature (*T*_C_), first melting temperatures (*T*_M1_), heat fusion (*Hf*), crystallinity, and MFR were measured as responses for the DOEs. The RSM will produce the statistical models for these responses, along with optimized values of variables for the maximization of individual responses. For that, the experiment was designed composing a set of 17 experimental runs. These experiments were separately performed for pure iPP and LMPP, and their blends. However, the pure iPP and LMPP were not included in design run. The effects of several independent input variables (iPP and LMPP blends) on the dependent output response variables (thermal and mechanical properties) will be investigated through the RSM approach. RSM will define the relationship between these several independent variables and response variables, by employing a series of experiments intended for an optimal response [20]. Also, it is beneficial for determination and prediction data of optimum mix proportions with a minimal number of experiments [12].

#### Model Development for Surface Response Methodology (RSM)

A common RSM design was employed for this work using a statistical software Design-Expert V11-Trial, released by Stat-Ease Inc., and also for ANOVA analysis [26]. Input variables (or parameter) are the various ratios between iPP and LMPP polymer with variant contents. For the empirical model, these independent variables, the blend ratios of two polymers (iPP and LMPP), were considered in weight parts or fraction [27], and iPP/LMPP blends were made and tested individually to set the upper and lower limit data for dependent responses in software. The blend ratios were taken from 5–25 weight parts for LMPP, and 75–95 weight parts for iPP at constant settings of blending. Whereas, from the knowledge of blending at various polymer content ratios as input parameters [1], the information of two cases of pure iPP and LMPP were segregated and thus eliminated. Aforementioned, thermal and tensile properties, namely elongation at breakage, tensile strength and elastic modulus, *T*_C_, *T*_M1_, *Hf*, and crystallinity were measured as RSM responses to predict and optimise thermal and mechanical properties of monofilament yarns.

The regression analysis between independent variables and the response was performed to fit the empirical second-order polynomial model and is shown in the following equation:

$$y=c_{0}+\sum_{i=1}^{N}c_{i}x_{i}+\sum_{i=1}^{N}c_{ii}x_{i}^{2}+\sum_{i,j=1;j<i}^{N}c_{ij}x_{i}x_{j}\;$$

where *c*_0_, *c_i_*, *c_ii_*, and *c_ij_* represent model parameters or regression coefficients in the intercept, linear, quadratic, and interaction terms, respectively. *x_i_* and *x_j_* are designed (independent) variables.

The experimental levels of independent variables and dependent responses are given in Table 2. As the experiment consists of 17 runs, to minimise the influence of uncontrolled parameters, the experimental sequence was randomised. The content ratios of iPP homopolymer and LMPP were taken as independent variables (x1 and x2). Parameters of elongation at breakage (y1), tensile strength (y2), elastic modulus (y3), crystallinity (y4), *T*_C_ (y5), *T*_M1_ (y6), MFR (y7), and *Hf* (y8) are the predicting responses. The adequacy of the built model was checked with ANOVA computations and various statistical parameters. Probability (*p* value), Fisher (f value), the correlation coefficient (R^2^), adjusted correlation coefficient (Adj R^2^) and predicted correlation coefficient (Pred R^2^) were used for adequate approximation and validity of model [17]. P and f values at 95% confidence level (*p* < 0.05) are significant for all of the predicted designs based on experimental data.

## 3. Results and Discussion

### 3.1. Regression Models Development

The present work deals with the concept of RSM with 17 experimental runs carried out to determine the optimum iPP/LMPP blending parameters. The statistical combination of independent variables and analysis of variance for the experimental results with their response are presented in Table 2. According to a famous statistician, George E. P. Box, “Models can be statistically unsound or erroneous but some can be useful” [21]. Meanwhile, to get the usefulness of RSM model, quadratic polynomials were used instead of cubic polynomials, as cubic models are often used for the overfitting of variations. Moreover, for the prediction of optimization of a system, it is important to evaluate the significance of parameters [25]. The types of models fitted for the experimental data here were mostly quadratic and 2Fl. Quadratic expressions were used for the development of the polynomial regression equations for elongation at break, elastic modulus, *T*_C_, *T*_M1_ and *Hf*; while for tensile property 2Fl, they were identified as significant by the software.

**Figure 1:**
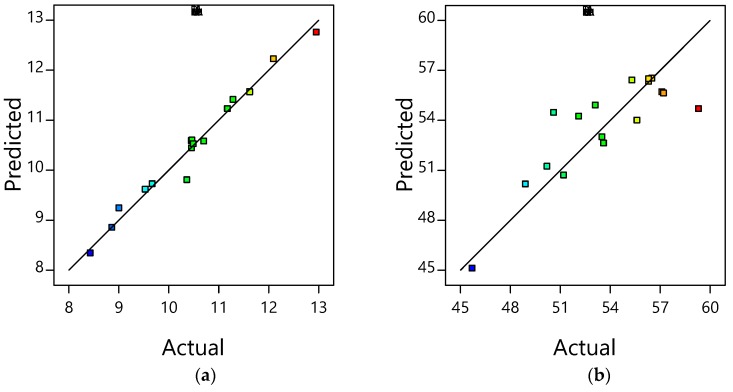

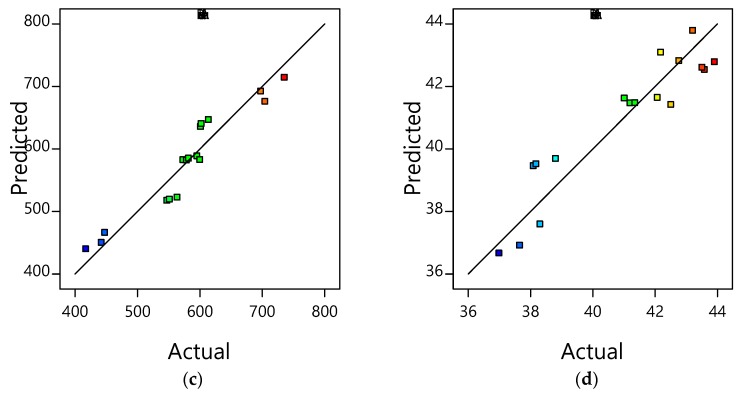
Plots of predicted versus actual values for: (**a**) elongation, (**b**) tensile strength, (**c**) elastic modulus, and (**d**) crystallinity.

**Figure 2:**
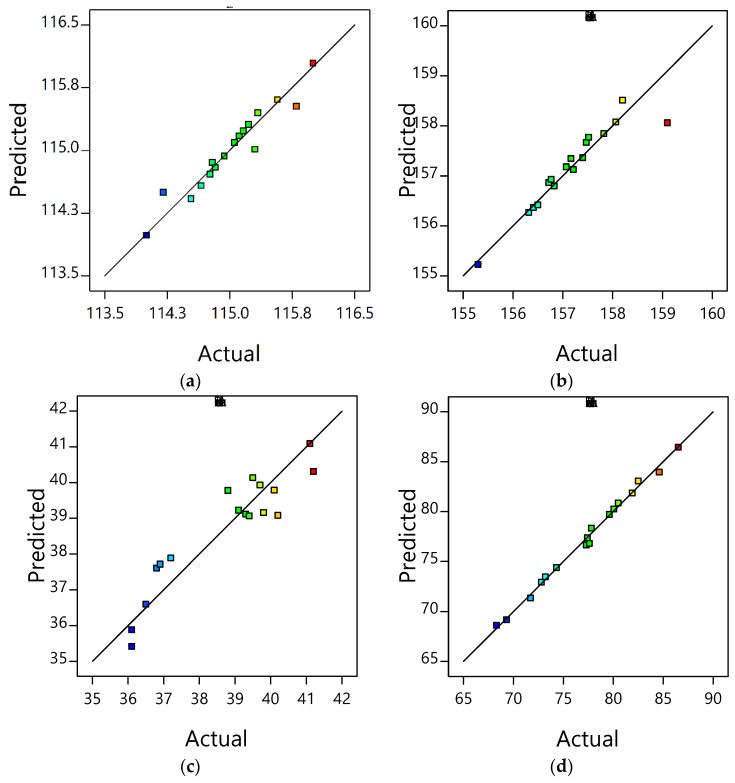
Plots of predicted versus actual values for: (**a**) crystallization temperature (*T*_C_), (**b**) first melting temperature (*T*_M1_), (**c**) melt flow rate (MFR) and (**d**) heat fusion (*Hf*).

The predicted response values from the RSM model and the actual values obtained from the experiments were analyzed as predicted versus actual plots that are shown in Figure 1 and Figure 2. It shows the actual iPP/LMPP blending activity and operational parameters corresponding to the predicted results from the established RSM empirical model. Also, from two figures, adequacy of the model can be determined based on the data points distributed around the mean of each variable responses. The more uniform the data points distributed near the mean of response variable, the more adequate the model will be [23]. Moreover, Figure 1 and Figure 2 shows the relationship between the predicted and actual values of each attributed thermal and mechanical property’s parameters. An adequate correlation to the linear regression fit can be seen in most of the response variable graphs. The average of R^2^ for elongation, elastic modulus, *T*_C_, *T*_M1_, MFR, crystallinity, *Hf*, and tensile strength are 0.976, 0.918, 0.938, 0.889, 0.867, 0.869, 0.995, and 0.718, respectively. Apart from the precision, the R^2^ values of each attribute were within the desirable range; the regression analysis was carried out by evaluating the adjusted R^2^ and predicted R^2^. The greater correlation coefficients further confirm the appropriateness of the formulated RSM models. Although the difference between the R^2^ values and adjusted R^2^ values for every response was evident, the R^2^ values were in conceivable agreement with the adjusted R^2^ values. They were 0.966 for elongation, 0.889 for elastic modulus, 0.905 for *T*_C_, 0.838 for *T*_M1_, 0.809 for MFR, 0.808 for crystallinity, 0.995 for *Hf* and 0.607 for tensile strength, respectively. Whereas the coefficient of variation percentage (C.V %) was less than 5% in all responses, indicating the established model is reproducible [13,28]. This implies that the model can be used for further parametric analysis of LMPP content in the influence of the properties, and to optimise the parameters ultimately.

### 3.2. Effects of LMPP on Thermal and Mechanical Properties

**Figure 3:**
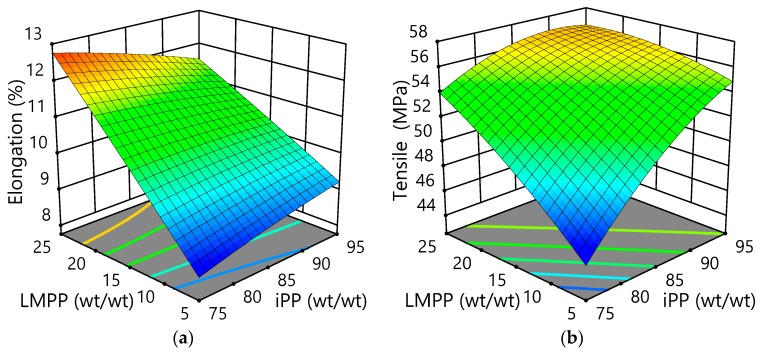

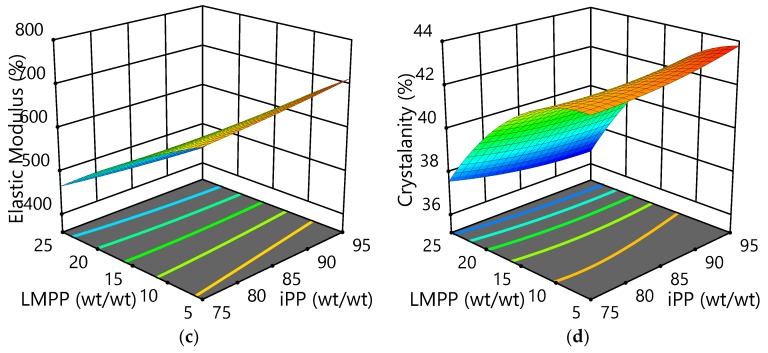
3D surface plots for the effects of LMPP on iPP/LMPP blend fibres for: (**a**) elongation, (**b**) tensile strength, (**c**) elastic modulus, and (**d**) crystallinity.

The generated 3D surface plots by RSM assist in visualizing the effect of parameters on a wide array of responses. Figure 3a–d shows 3D surface response plots for the effects of variant LMPP contents on the properties of iPP/LMPP blend fibres. In order to investigate and find the optimized thermal and mechanical properties with different iPP/LMPP ratios, it is required to observe the structural development of iPP/LMPP blend fibres (discussed below). Figure 3a,b shows that, with the certain amount of LMPP contents, the mechanical properties have been improved dramatically. It can be seen from Table 2, that the pure iPP fibre has the lowest elongation at break and highest elastic modulus. Therefore, it is important to understand the iPP/LMPP parameters individually. The percent elongation at break, which partly reflects the extent stretching, was found improved with the addition of LMPP to the blend fibres, as can be seen in Figure 3a. However, it is shown in Figure 3b, that the tensile properties were not affected appositely with the increased ratio of LMPP. It has been reported that the maximum stress and strain rate was decreased with an increased ratio of LMPP [29].

Likely, the elastic modulus of iPP/LMPP blend fibres diminishes with the increase of LMPP content, as shown in Figure 3c, which demonstrates the fibre’s smoothness and softness. This is due to the unique stretchability and low modulus of LMPP; moreover, the modulus of iPP/LMPP fibres is affected predominantly by crystallinity in crystalline polymers and molecular orientation [1]. Considering the advantages in fibre spinning applications, narrow molecular weight distribution with relatively low molecular weight is essential. Whereas, for thick sheets and extrusion of pipes, where high strength is required, relatively broad molecular weight distribution and high molecular weight are essential [30].

In Figure 3d it can be observed that the crystallinity of iPP/LMPP blends reduce with the increase of LMPP, but reach an inelastic peak at a certain point. This drastic change in the fibre’s structure is due to the addition of LMPP into the amorphous phase of the PP lamella structure [4]. Moreover, it has also been reported that due to the slower crystallization speed of LMPP, the blending of LMPP to iPP reduces the crystallization speed and the spherulite growth speed, and hence the blend structure [4]. Likewise, the crystallization of pure iPP occurrs in a spin-line, normally at higher temperatures prior to pure LMPP, which also affects the iPP/LMPP blends’ structural orientation [31,32]. It is interesting to see in Figure 3d, a slight plateau in relation to the crystallinity with higher LMPP content blends, which indicates that the addition of LMPP content beyond a level will not optimise the system (fibre blend). As there is variation in such levels in predicted RSM models with similar surface responses, this does not affect the studied systems [25]. In addition, it also shows a drastic change in the thermal property; the crystallinity reduces suddenly with an increase of LMPP. However, specific weight fractions of LMPP (5–10 wt) have been found to improve the crystalline perfection and crystalline uniformity of iPP, increasing the spinnability of the blend fibres [1]. This phenomenon can help in producing nonwovens with enhanced softness, fineness and uniformity [1,29].

**Figure 4:**
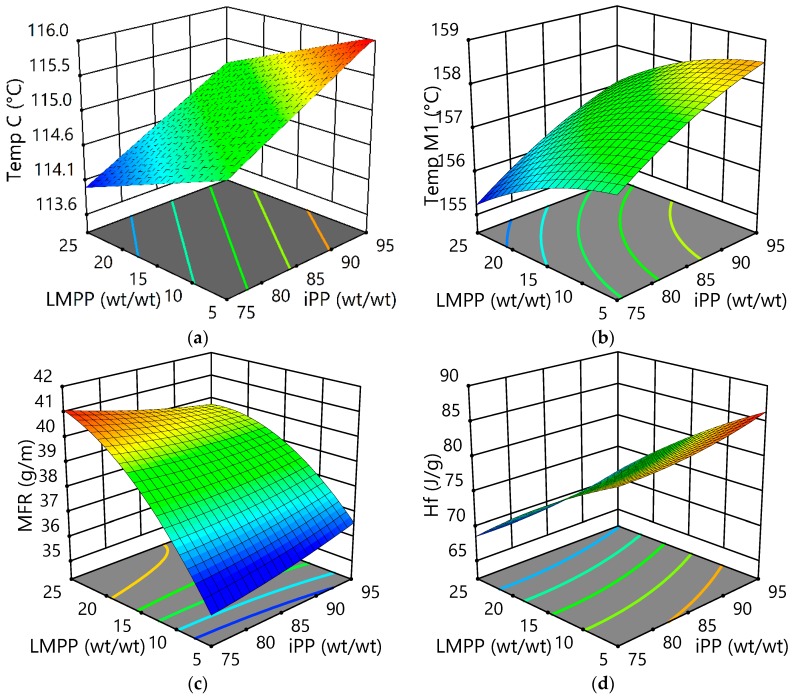
3D surface plots for the effects of LMPP on iPP/LMPP blend fibres for (**a**) crystallization temperature (*T*_C_), (**b**) first melting temperature (*T*_M1_), (**c**) melt flow rate (MFR) and (**d**) heat fusion (*Hf*).

Very often it is difficult to measure the *T*g and *T*_M_ of iPP fibre and its fibre blend accurately, which is caused by the weak transition in its molecule structure. The higher the temperature, the greater the molecular motion will be. The freedom of molecular motion makes the crystallization difficult at a given temperature, thus increasing the time needed for crystallization [1]. The addition of LMPP delays the crystallization rate when blended with homo PP, as a result, the mold of formability and transferability is attained. In other words, the addition of LMPP enhances the stretchability and spinnability of the blend fibres. From Figure 4a, it can be seen that with LMPP content increase, the crystallization temperatures of iPP/LMPP blend fibres decrease. Figure 4c shows a visible change in MFR with the addition of LMPP into iPP. Figure 4b–d presents the overall *T*_M1_ and *Hf* measured. *Hf* values reduce with the content of LMPP increased in iPP/LMPP blend fibres. The apparent change of *Hf* values reflects the melting behavior of iPP itself within its structure formed with the addition of LMPP. Hence, change in *Hf* affects the overall crystallinity of the blends [33]. It can be seen from Table 1 that the melting point of pure LMPP is lower than that of iPP, due to its lower isotacticity. On the other hand, with variant LMPP content in the fibre blend, the melting temperatures of iPP/LMPP blend fibres change slightly, especially the *T*_M1_ [4]. This increase in melting behaviors of iPP/LMPP blend fibres also indicates that a small amount of LMPP molecules are incorporated into the iPP crystals [31,32].

Generally, polymers with high molecular weight have stronger intermolecular interaction, which causes low MFR and higher viscosity [34]. Whereas, polymers with high MFRs often feature low molecular weight or broad molecular weight distribution [8]. Changes in MFR values of polymers influence the properties of fibres, such as elongation and bond strength. Fibres with lower MFR values have higher bond strength at lower temperatures due to the molecular effect. As for the MFR analysis in this study, pure LMPP was found to have a higher MFR value compared to pure iPP and all other fibre blends, as shown in Figure 4c. With the increase in the weight fraction of LMPP, the MFR of iPP/LMPP blend fibres also increased. For the fibre blends with the greater LMPP content, the strain rate during the spinning of fibres was increased due to the increased fluidity of molten polymer blend, and as a result, the produced blend fibres were finer under the same spinning parameters [17]. This phenomenon suggests the improvement in spinnability of fibre blends, which may be due to a decrease in stereo-regularity of LMPP [1,31,32].

### 3.3. Optimization of Thermal and Mechanical Responses 

A dramatic change in mechanical properties of iPP/LMPP blends is anticipated if the auxiliary polymer is out of the tested proportion. Likewise, it is considered reasonable to blend iPP and atactic polypropylene (aPP) for their similar molecular structure and good affinity between them [35,36], but at a cost of serious degradation to the mechanical properties of iPP products [35]. The content above 20 wt % of aPP in iPP/aPP blends, was found to alter the mechanical properties drastically due to percolation phenomenon [37]. Likewise, it was also reported that the mechanical properties of iPP fibre degraded with the addition of aPP component. The increased impact strength of iPP/aPP blend was found to be achieved with 5 to 20 wt % of aPP content addition, however, with a decrease in yield strength from about 40 MPa to 20 MPa [38]. Similarly, a change in thermal properites of iPP blends was found to alter with the addition of aPP to them, for instance *T*_M_, *Hf* of melting, *T*_C_, and latent heat of crystallization, were found to decrease evidently in all blends with increasing aPP content [39]. Hence, an appropriate ratio of the auxiliary polymers is crucial when blending with iPP.

Generally, batch and semi-batch reactors are utilised for the production of high-value polymers, for which the optimal recipe is essential. To achieve the optimal recipe, a number of experiments are carried out as a trial and error method. This trial and error method not only is used to optimize operating conditions but also to make assumptions by monitoring the influence of one factor at a time on an experimental response, whereas only one parameter is altered and others are kept constant [40]. In addition, exhaustive experimental repetition is required, including loading conditions, temperature profiles, and material and resource wastage, especially for blended polymers to get the ideal blend ratio. To cope with such an issue, the statistical design of RSM can be employed, comprising influences of individual factors including their interactive influences, by the fitting of a polynomial equation to the experimental data [22]. As mentioned before, designing an experiment model with RSM helps in evaluating the effects of various factors, achieving optimum conditions and reducing the number of experiments [12,13].

In order to justify the optimization of individual mechanical and thermal properties obtained in Figure 3 and Figure 4, the response optimiser part of the Design-Expert Software was used. The software offers preferences that are “minimum”, “maximum”, “target” and “in range” for the dependent and independent variables. Moreover, there is another preference to the above-mentioned one that is “equal to” for the independent variables [23]. The optimization of independent and dependent variables employed in this study was achieved by the preferences of “in range” and “maximum”. For a better understanding of the independent and dependent variables, three polymer blend ratios of iPP/LMPP were chosen, which are for maximum mechanical and thermal properties, and for optimised properties of combined mechanical and thermal. Three supplementary experiments were carried out. The results are presented in Table 3. The maximum values for mechanical properties i.e., tensile strength, elongation at break and elastic modulus from the optimised iPP/LMPP blend ratio of 77.5 iPP and 22.5 LMPP weight parts were obtained via software modeling. Whereas, the maximum values for thermal properties i.e., crystallinity, the heat of fusion and MFR were obtained at 91.2 wt iPP/9.8 wt LMPP.

**Figure 5:**
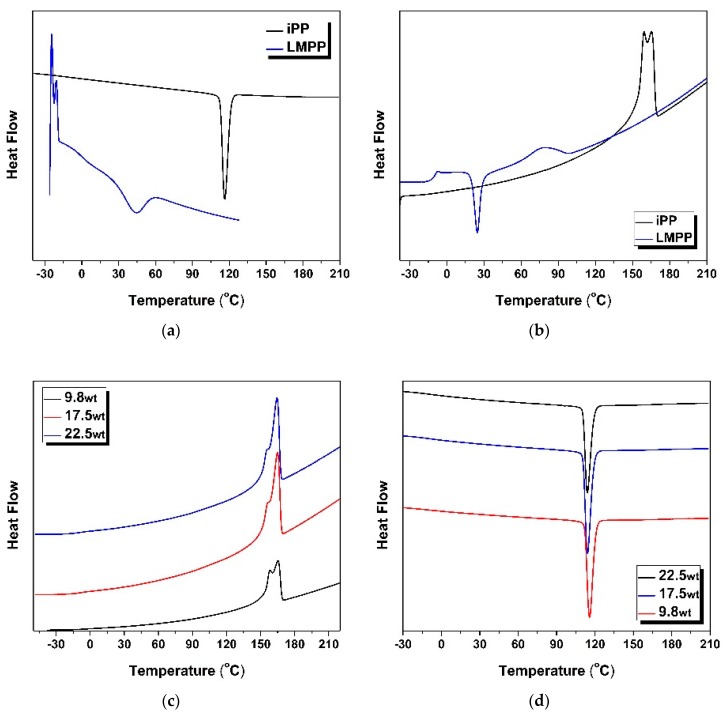
DSC curves with (**a**) second-heating curve and (**b**) second-cooling curve for pure iPP and LMPP, including (**c**) second-heating curve and (**d**) second-cooling curves for iPP/LMPP blends percentage suggested by the RSM model.

The changes in thermal properties of predicted iPP and LMPP blend ratios are shown in Table 3, from which DSC values, crystallization and melting behaviour were investigated. Results of the blends along with pure iPP and LMPP are shown in Figure 5a–d. The *T*g of pure LMPP was obvious around −10.2 °C, while for the pure iPP was not, as seen from the DSC of heating curves in Figure 5a,b. A similar trend was seen for the blends in Figure 5c,d. This transitional change in blends is due to the high crystallinity and crystallization rate of iPP and eventually in fibres when blended with LMPP. Moreover, the amorphous part is too limited for the *T*g signals to be detected by DSC. On the other hand, LMPP has a low crystallization rate and low crystallinity, which can also be confirmed by its cold-crystallization peak at about 24.5 °C. This re-crystallization process of LMPP is carried out when the molecular chain gets more dynamic in the relatively high temperatures [1]. No obvious cold-crystallization peaks were seen in the pure iPP DSC curve in Figure 5a,b and in the iPP/LMPP blends in Figure 5c,d.

There were changes in the DSC curves among three predicted blend ratios by RSM, but some similarities and differences were seen as well in their melting peak trends. For instance, in Figure 5b, double melting peaks, which is due to the imperfect crystalline that is subjected to the melting re-crystallization process and the second melting peak temperature is the same at about 165.5 °C. Moreover, compared with pure iPP, the iPP/LMPP blends showed improved crystalline uniformity, due to lower molecular weight of LMPP. Aiming at the iPP/LMPP blend fibres with better thermal properties, the predicted blend ratio 9.8 wt showed a lower melting peak compared to the other two ratios, 22.5 and 17.5 weight parts of LMPP. As pure LMPP has a low and wide melting peak, indicating low crystallinity from iPP, this makes it useful in the processability of the polymer manufacturing. However, change in melting trends such as *T*g may affect MFR, as well as the flow-ability of a polymer [41]. On one hand, higher LMPP content in the blended fibres demonstrated enhanced mechanical attributes; however, with increased LMPP contents, the crystallization temperature and the crystallization entropy value of iPP/LMPP blends was found to decrease, suggesting moderate usage of LMPP contents for desired or optimal thermal properties.

**Figure 6:**
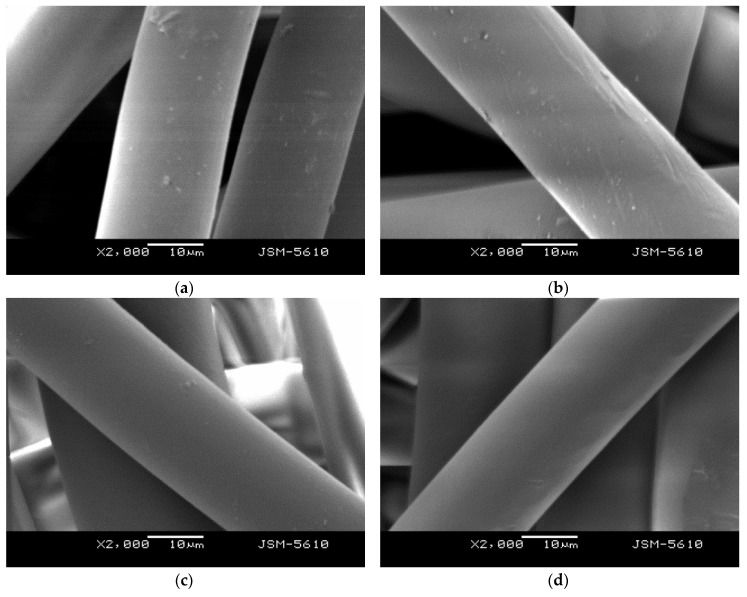
SEM images of the iPP/LMPP blend fibres with (**a**) 9.8 wt LMPP, (**b**) 17.5 wt LMPP and (**c**) 22.5 wt LMPP percentage suggested by the RSM model and (**d**) pure iPP.

The SEM images of RSM suggested iPP/LMPP blends with variant LMPP contents are shown in Figure 6a–d. A slight variation in the surface structures is visible. The SEM image of pure iPP in Figure 6d appears smoother in contrast to 9.8 wt of LMPP content blend fibres in Figure 6a, which is obvious because of their different morphology and crystallinity. A good miscibility is apparent in all the iPP/LMPP blends, for the required functionality of the fibres, variant LMPP contents can be used.

Optimization factors to achieve fibre blend with desired properties were determined from appropriate spinnability with better mechanical and thermal property perspectives. A blend ratio of 82.5 wt iPP and 17.5 wt LMPP was suggested by the developed RSM model. The accordance between the predicted and the actual results with a marginal difference were in the permissible limits, indicating the blend ratios obtained from RSM were practical for attaining optimized mechanical and thermal properties in fibres. It has been approved that the functionality and processability of the blend fibres can be improved with the addition of LMPP into iPP, if correctly proportioned.

## 4. Conclusions

Response surface methodology (RSM) was used to study the effects of blend polymerization factors of isotactic polypropylene (iPP) and low molecular low modulus polypropylene (LMPP) on the mechanical and thermal behavior of iPP/LMPP blend fibres through a Design-Expert software analysis. Empirical models to simulate the variant blend ratios of iPP/LMPP were developed in order to optimie the output responses of the blend fibres. The models were validated by experimental results, and a satisfactory correlation coefficient was achieved for the evaluated models. Variation in the mechanical and thermal properties was seen by changing LMPP contents from 5 to 25 weight parts in iPP/LMPP blend fibres. Fibres with lower LMPP content showed higher values for elastic modulus (MPa), crystallinity (%), heat fusion (J/g), crystallization and overall melting temperature (°C). Whereas, higher values for tensile strength (MPa), elongation at break (%) and melt flow rate (g/10 min) were obtained in higher LMPP content blend fibres. A blend ratio of 82.5 wt iPP and 17.5 wt LMPP was suggested by the RSM model, to achieve desired properties determined from appropriate spinnability with better mechanical and thermal property perspectives. The results were approved by the experimental results, indicating the validity and reliability of the developed RSM model. This implies that the developed RSM model can be used to predict/estimate the properties of fibre blends to inform industrial practice.

## Figures and Tables

**Table 1:** Molecular characteristics of the iPP and LMPP sample.

Materials	Type	Catalyst	*M*_w_^1^	*M*_w_/*M*_n_^2^	Density (kg/m^3^)	MFR ^3^ (g/10 min)	*T*_M_^4^ (°C)
iPP	Isotactic	Ziegler Natta	200,000	3.5	900	36	165.5
LMPP	Atactic	Metallocene	130,000	2	870	50	79.1

^1^*M*_w_: Molecular Weight; ^2^*M*_w_/*M*_n_: Molecular Weight/Molecular Number; ^3^ MFR: Melt Flow Rate; ^4^ *T*_M_: Melting Temperature.

**Table 2:** The design of experiments and responses for iPP/LMPP filaments.

Run	iPP (*wt*/*wt*)	LMPP (*wt*/*wt*)	Elongation (%)	Tensile Strength (MPa)	Elastic Modulus (MPa)	Crystallinity (%)	Crystallization Temperature-*T*_C_ (°C)	First Melting Temperature-*T*_M1_ (°C)	Melt Flow Rate-MFR (g/10 min)	Heat Fusion-*Hf* (J/g)
1	75	5	8.4	45.7	704.1	42.7	114.7	156.8	36.1	82.4
2	90	10	10.3	59.3	613.5	43.9	115.8	159.1	37.2	81.9
3	90	15	10.4	57.1	581.5	41.3	115.2	157.5	40.2	77.8
4	85	25	12.0	56.3	441.8	37.6	114.6	156.4	41.2	69.3
5	80	15	10.4	53.6	578.2	41.1	114.7	156.7	39.3	77.6
6	75	15	10.4	48.9	572.7	41.0	114.5	156.3	39.1	77.3
7	80	10	9.5	51.2	600.9	43.5	114.9	157.0	36.8	80.0
8	95	25	11.6	56.5	416.9	36.9	115.1	157.2	38.8	71.7
9	85	5	8.8	50.2	697.4	42.1	115.3	157.8	36.1	84.6
10	85	10	9.6	53.5	601.9	43.5	115.1	157.4	36.9	80.5
11	90	20	11.1	55.3	547.0	38.0	115.0	157.1	40.1	74.3
12	85	20	11.2	57.2	551.0	38.1	114.8	156.7	39.7	73.2
13	85	15	10.7	50.6	599.7	42.5	115.3	157.4	39.4	77.4
14	95	5	9.0	53.1	735.2	43.2	116.0	158.2	36.5	86.5
15	95	15	10.4	56.3	595.1	42.0	115.5	158.0	39.8	79.6
16	75	25	12.9	55.6	447.3	38.3	114.0	155.3	41.1	68.3
17	80	20	11.6	52.1	563.4	38.8	114.2	156.5	39.5	72.8
* C	100	0	2.2	52.8	753.7	40.2	116.3	159.1	34.8	91.4
* C	0	100	9.5	25.3	89.4	16.5	40.1	79.1	49.9	20.6

* Pure LMPP and iPP are not included in design run.

**Table 3:** Solutions for iPP/LMPP optimum conditions for blend fibres with desired properties.

Experiment	iPP (*wt*/*wt*)	LMPP (*wt*/*wt*)	Elongation (%)	Tensile Strength (MPa)	Elastic Modulus (MPa)	Crystallinity (%)	Crystallization Temperature-*T*_C_ (°C)	First melting Temperature-*T*_M1_ (°C)	Melt Flow Rate-MFR (g/10 min)	Heat Fusion-*Hf* (J/g)
1-Predicted	77.5	22.5	12.15	53.33	519.70	37.97	114.24	155.93	40.37	70.77
1-Actual	77.5	22.5	12.90	54.85	510.10	38.23	114.10	155.23	41.56	68.90
2-Predicted	91.2	9.80	09.78	53.15	616.31	43.99	115.61	158.11	37.52	82.30
2-Actual	91.2	9.80	10.59	55.43	614.24	43.23	115.31	157.91	37.12	81.99
3-Predicted	82.5	17.5	11.00	51.98	576.36	39.90	114.70	165.05	39.25	75.15
3-Actual	82.5	17.5	10.79	50.51	597.94	42.12	115.24	159.96	39.51	77.01
